# Chronic insomnia, high trait anxiety and their comorbidity as risk factors for incident type 2 diabetes mellitus

**DOI:** 10.1038/s41598-024-62675-y

**Published:** 2024-05-24

**Authors:** Pauline Duquenne, Cécilia Samieri, Stéphanie Chambaron, Marie-Claude Brindisi, Emmanuelle Kesse-Guyot, Pilar Galan, Serge Hercberg, Mathilde Touvier, Damien Léger, Léopold K. Fezeu, Valentina A. Andreeva

**Affiliations:** 1 INSERM, INRAE, CNAM, Center for Research in Epidemiology and Statistics (CRESS), Nutritional Epidemiology Research Team (EREN), SMBH, Sorbonne Paris Nord University and Paris Cité University, 74 Rue Marcel Cachin, 93017 Bobigny, France; 2 Bordeaux Population Health, INSERM U1219, University of Bordeaux, Bordeaux, France; 3 Center for Taste and Feeding Behavior, CNRS, INRAE, Agro Dijon Institute, University of Burgundy, Dijon, France; 4 University Hospital Center, Dijon, France; 5 Department of Public Health, AP-HP Paris Seine-Saint-Denis Hospital System, Bobigny, France; 6 Université Paris Cité, VIFASOM, APHP Hôtel-Dieu, Centre du Sommeil et de la Vigilance, Paris, France; 7 Division of General Medicine, Department of Medicine, Columbia University Irving Medical Center, New York, NY USA

**Keywords:** Anxiety, Comorbidity, Diabetes mellitus, Insomnia, Mental health, Prospective study, Anxiety, Diabetes

## Abstract

The main objective of this study was to evaluate the association of the insomnia-anxiety comorbidity with incident type 2 diabetes (T2D) in a large prospective cohort. We selected adults without diabetes at baseline from the French NutriNet-Santé cohort who had completed the trait anxiety subscale of the Spielberger State-Trait Anxiety Inventory (STAI-T, 2013–2016) and a sleep questionnaire (2014); insomnia was defined according to established criteria. Using multivariable Cox models, we compared T2D risk across 4 groups: no insomnia or anxiety (reference), insomnia alone, anxiety alone (STAI-T ≥ 40), and comorbid anxiety and insomnia. Among 35,014 participants (mean baseline age: 52.4 ± 14.0 years; 76% women), 378 (1.1%) developed T2D over a mean follow-up of 5.9 ± 2.1 years. Overall, 28.5% of the sample had anxiety alone, 7.5%—insomnia alone, and 12.5%—both disorders. In the fully-adjusted model, a higher T2D risk was associated with anxiety-insomnia comorbidity (HR = 1.40; 95% CI 1.01, 1.94), but not with each disorder separately, compared to the group without insomnia or anxiety. The findings supported a positive association between anxiety-insomnia comorbidity and incident T2D among general-population adults. Future research using clinical diagnoses of mental disorders could confirm the findings and guide diabetes prevention programs.

## Introduction

Diabetes mellitus represents a major public health challenge owing to its prevalence, chronicity and deleterious impact on various organs and tissues^1,2^. Type 2 diabetes (T2D), which accounts for 90% of all diabetes cases, has multifactorial etiology, including genetic, psychosocial, behavioral, and psychiatric causes^1^. It is therefore critical to advance knowledge about modifiable factors in order to better target primary prevention of the disease.

A recent umbrella review provided strong support for the increased prevalence of T2D among individuals with psychiatric conditions, particularly anxiety and insomnia^3^. Moreover, comorbidity, defined as the simultaneous presence of ≥ 2 conditions in the same individual, of these two mental disorders is also frequent^4^. Anxiety is underscored by anticipation of a future threat and presents symptoms such as agitation, tiredness, difficulties concentrating, irritability, and muscle tension^5^. Insomnia—the most prevalent sleep disorder—is defined by a difficulty falling asleep, maintaining sleep or early morning waking, with such symptoms occurring ≥ 3 nights/week over the past ≥ 3 months^5^. According to the most recent estimates by the French national public health agency, the general population prevalence of anxiety and insomnia was 25% and 13%, respectively^6,7^.

A systematic review of observational studies reported that elevated symptoms of anxiety were present in 40% of individuals with diabetes^8^. Further, one meta-analysis of 14 longitudinal studies with a follow-up ranging from 2 to 14 years reported a 47% increased risk of incident T2D associated with baseline anxiety^9^. It included studies with heterogeneous methodologies featuring different types of anxiety disorders (e.g., generalized anxiety disorder, panic disorder, phobic disorders, post-traumatic stress disorder)^9^.

On the other hand, numerous prospective studies have investigated the association between various sleep disorders and T2D; the findings have been relatively consistent and summarized in several meta-analyses^10–13^. Prior research has included different symptoms or types of sleep disorders, such as acute insomnia, difficulty falling asleep, non-restorative sleep, and short total sleep time without considering the duration or frequency of experiencing the symptoms. Some studies have focused on insomnia (without considering its impact on daily life) in different general and specific (i.e., pre-diabetic) populations and have found positive associations with incident T2D^14–16^.

To our knowledge, the anxiety-insomnia comorbidity has not been investigated as a predictor of incident T2D in an epidemiological context. However, anxiety and insomnia form a vicious circle and share many biological mechanisms^17^. They also share several pathways affecting the central nervous system and metabolism, and may represent mechanisms for the development of T2D^17^. Thus, our objective was to evaluate the association of anxiety, insomnia and their comorbidity with incident T2D among adults. We hypothesized that individuals with anxiety-insomnia comorbidity would have a greater risk of developing T2D than would individuals with anxiety or insomnia alone or those without either disorder.

## Materials and methods

### Study overview

This prospective analysis is part of the 4-year multidisciplinary MEMORIES project focused on mental-physical comorbidity. Specifically, the associations between a number of individual and concurrent mental health conditions (e.g., anxiety, insomnia, depressive symptoms, eating disorders) and metabolic disorders (notably T2D and obesity) are investigated among general-population adults in order to advance knowledge about risk factors and primary prevention options.

### Study population

NutriNet-Santé is an ongoing web-based prospective cohort launched in 2009 in France. Its main aims are: (1) to elucidate the relationship between nutrition, health status, chronic morbidity, and mortality, and (2) to examine the determinants of dietary habits and nutritional status. The cohort’s design and methods have been described elsewhere^18^. Eligible participants are men and women aged ≥ 18 years, with desktop or mobile Internet access and able to follow an online protocol in French. They are recruited from the general population primarily via recurrent multimedia campaigns.

Once enrolled, participants complete online (https://etude-nutrinet-sante.fr/) a set of 5 questionnaires related to their sociodemographic and anthropometric characteristics, lifestyle choices, physical activity and sedentariness, physical and mental health status, and dietary intake.

### Data collection

#### Type 2 diabetes mellitus assessment

Health status is self-reported at enrollment and is reassessed every 6 months over the follow-up, using a specifically developed health status questionnaire. At any time during the follow-up, participants can also report a major or minor health event, a new prescribed treatment, a medical exam or a hospitalization via a dedicated and secure online platform. T2D cases were defined using ICD-10 code E11 and ATC classification codes for T2D medication. Moreover, in NutriNet-Santé, all self-reported diagnosis and medication use data are linked to the national health insurance database (SNIIRAM), providing information about reimbursement of prescribed medication and hospitalizations. For this study, we considered all incident cases occurring between inclusion and April 2022. Next, T2D mortality cases were identified using a linkage to CépiDC, the French national mortality registry. Details about case ascertainment are provided in Supplementary Table S1. T2D incidence was the main outcome in this study.

#### Trait anxiety assessment

Trait anxiety was one of the two main exposures in this analysis. It was evaluated by self-reports on the trait anxiety subscale of the State-Trait Anxiety Inventory Form Y (STAI-T). The STAI is one of the most widely used tools for assessing anxiety, particularly as it distinguishes it from depression^19^. The French version of STAI has been validated in adults from the general population^19^. STAI-T consists of 20 items (e.g. "I worry too much about things that are not worth it"), scored on a 4-point Likert scale ranging from "Almost never" to "Almost always". The total score range is 20–80 points; the higher the score, the higher the anxiety proneness. Participants were considered as having high trait anxiety if their STAI-T score was ≥ 40^20^. Anxiety as measured by the STAI-T is highly correlated with generalized anxiety disorder^21^. In NutriNet-Santé, this questionnaire was administered between November 2013 and December 2016 to participants enrolled in the cohort for ≥ 2.5 years, which represented a total of 119,451 enrollees solicited. Each participant was allowed to complete it once, on a voluntary basis. The STAI-T completion date was considered as baseline for this analysis.

#### Insomnia assessment

Chronic insomnia was also one of the main exposures in this analysis. A questionnaire assessing sleep habits and characteristics was administered between February and July 2014 to all participants of NutriNet-Santé at that time. To define chronic insomnia, we followed the International Classification of Sleep Disorders—Third Edition (ICSD-3)^22^ and the Diagnostic and Statistical Manual of Mental Disorders—Fifth Edition (DSM-5)^5^. For chronic insomnia the criteria include experiencing sleep problems (difficulties falling asleep, frequent nighttime waking), ≥ 3 nights/week over the past ≥ 3 months, and experiencing negative repercussions of such problems in daily life.

#### Covariate data collection

Socio-demographic data including sex, age, education (< high school, high school diploma, college/undergraduate degree, graduate degree), employment category (manual/blue collar/farmer, office work/administrative staff, professional/executive staff, retired, without professional activity), children < 18 years in the household (yes/no), and lifestyle data including smoking status (never, former or current smoker) and alcohol use (g ethanol/day) are provided by a validated self-reported questionnaire^23^. Physical activity (low, moderate, high) and sedentariness (minutes spent sitting/day) were assessed with the International Physical Activity Questionnaire using established scoring guidelines^24^. Anthropometric data (height and weight) were self-reported using a validated questionnaire^25^. Data on hypertension, dyslipidemia, gestational diabetes and family history of diabetes were obtained from the health status questionnaire. As all of the above-mentioned questionnaires are administered on an annual basis, we used data provided within a 2.5-year window around baseline.

Depressive symptomatology was also modelled as a covariate; it was assessed with the Center for Epidemiologic Studies-Depression Scale (CES-D)^26,27^, which is administered every 2 years during the follow-up. This is a 20-item questionnaire where participants report their experience of symptoms associated with depression over the past week; the items are rated on 4-point scale ranging from 1 = “Rarely or never” to 4 = “Most or all the time”. Higher scores indicate higher depressive symptomatology. The internal consistency assessed by Cronbach’s alpha coefficient was high (> 0.80) at each CES-D assessment. Participants were considered as having depressive symptoms if their CES-D score was ≥ 17 (for men) or ≥ 23 (for women)^26^, corresponding to the validated cut-offs for the French population.

Finally, mean energy intake (kcal/day) and proportion of energy from carbohydrates were assessed using a set of three 24-h dietary records which participants were asked to complete every 6 months. We selected CES-D and dietary data within the 2.5-year window around baseline.

### Statistical analyses

For the following covariates with < 5% missing values, we used multiple imputation techniques: education (4.0% missing values), employment (4.5%), alcohol consumption (3.2%), sedentariness (4.6%), smoking (3.2%), physical activity (4.5%) and depressive symptoms (3.0%). There were no covariates with > 5% missing values. Body mass index (BMI, kg/m^2^) was calculated using the self-reported weight and height data. The BMI values were then divided into four categories: underweight (< 18.5), normal weight (18.5–24.9), overweight (25.0–29.9), and obesity (≥ 30.0). For the analyses, morbidity and comorbidity groups were created as follows: neither anxiety nor insomnia, anxiety alone, insomnia alone, and comorbid anxiety and insomnia. Descriptive characteristics according to these morbidity groups are presented in Table 1, showing the number (%) and *p*-values of the chi-squared tests for categorical variables, and the mean values (± SD) and *p*-values obtained by ANOVA for continuous variables.

**Table 1:** Baseline characteristics^a^ of participants according to morbidity group (Etude NutriNet-Santé, France).

	Full sample	No anxiety or insomnia	Anxiety only^b^	Insomnia only	Anxiety-insomnia comorbidity	*p*-value^c^
N	35,014	18,027 (51.5%)	9986 (28.5%)	2615 (7.5%)	4386 (12.5%)	
Incident T2D	378 (1.1)	180 (1.0)	66 (1.1)	27 (1.0)	105 (1.5)	0.034
Age, years (mean, SD)	52.4 (14.0)	53.8 (14.0)	49.7 (14.5)	54.4 (12.3)	51.2 (12.9)	< 0.001
Age category						
18–34 years	5303 (15.1)	2378 (13.2)	2063 (20.7)	234 (8.9)	628 (14.3)	< 0.001
35–54 years	13,041 (37.2)	6270 (34.8)	3921 (39.3)	965 (36.9)	1885 (43.0)	
55–64 years	8808 (25.2)	4571 (25.4)	2188 (21.9)	855 (32.7)	1194 (27.2)	
≥ 65 years	7862 (22.5)	4808 (26.7)	1814 (18.2)	561 (21.5)	679 (15.5)	
Sex						
Male	8337 (23.8)	5442 (30.2)	1785 (17.9)	499 (19.1)	611 (13.9)	< 0.001
Female	26,677 (76.2)	12,585 (69.8)	8201 (82.1)	2116 (80.9)	3775 (86.1)	
Educational level^d^						
Less than high school	5005 (14.9)	2588 (14.9)	1401 (14.6)	368 (14.6)	648 (15.5)	< 0.001
High school diploma	5640 (16.8)	2904 (16.7)	1581 (16.5)	430 (17.1)	725 (17.3)	
Some college, undergraduate degree	10,672 (31.7)	5396 (31.1)	3020 (31.6)	841 (33.4)	1415 (33.8)	
Graduate degree	12,308 (36.6)	6460 (37.2)	3567 (37.3)	877 (34.9)	1404 (33.5)	
Employment category^e^						
Manual/blue collar, farmer	883 (2.6)	466 (2.7)	242 (2.5)	58 (2.3)	117 (2.8)	< 0.001
Office work/administrative staff	9963 (29.8)	4599 (26.6)	3161 (33.3)	748 (29.8)	1455 (35.1)	
Professional/executive staff	7869 (23.4)	4104 (23.7)	2254 (23.7)	540 (21.5)	931 (22.4)	
Retired	11,491 (34.4)	6834 (39.5)	2668 (28.1)	913 (36.4)	1076 (25.9)	
Without professional activity	3267 (9.8)	1278 (7.4)	1171 (12.3)	249 (9.9)	569 (13.7)	
Children aged < 18 years living in household (yes)	8954 (25.6)	4326 (24.0)	2785 (27.9)	623 (23.8)	1220 (27.8)	< 0.001
BMI (kg/m^2^) (mean, SD)	24.0 (4.4)	24.0 (4.1)	23.8 (4.5)	24.3 (4.4)	24.2 (5.0)	< 0.001
BMI category						
Underweight (< 18.5)	1625 (4.6)	656 (3.6)	588 (5.9)	107 (4.1)	274 (6.2)	< 0.001
Normal weight (18.5–24.9)	21,807 (62.3)	11,384 (63.1)	6262 (62.7)	1579 (60.4)	2582 (58.9)	
Overweight (25.0–29.9)	8495 (24.3)	4571 (25.4)	2244 (22.5)	668 (25.5)	1012 (23.1)	
Obese (≥ 30.0)	3087 (8.8)	1416 (7.9)	892 (8.9)	261 (10.0)	518 (11.8)	
Physical activity level^f^						
Low	7662 (22.9)	3340 (19.8)	2527 (26.6)	568 (22.8)	1137 (27.5)	< 0.001
Moderate	13,574 (40.6)	6892 (39.9)	3998 (42.1)	1053 (42.2)	1631 (39.5)	
High	12,186 (36.5)	6964 (40.3)	2981 (31.4)	875 (35.1)	1366 (33.0)	
Sedentariness (minutes spent sitting/day)^f^ (mean, SD)	348.8 (200.0)	337.5 (198.9)	364.4 (206.5)	339.9 (189.6)	365.2 (191.9)	< 0.001
Alcohol use (g ethanol/day)^g^ (mean, SD)	7.0 (9.8)	7.46 (10.0)	6.4 (9.3)	7.3 (10.0)	6.4 (9.7)	< 0.001
Smoking status^h^						
Never smoker	17,106 (50.5)	8775 (50.2)	5132 (53.2)	1179 (46.5)	2020 (47.9)	< 0.001
Former smoker	13,117 (38.7)	6993 (40.0)	3382 (35.1)	1098 (43.3)	1644 (39.0)	
Current smoker	3665 (10.8)	1728 (9.9)	1127 (11.7)	260 (10.2)	550 (13.1)	
Depressive symptoms over past week (yes)^i^	4007 (11.8)	343 (2.0)	1974 (20.5)	112 (4.4)	1578 (37.2)	< 0.001
History of hypertension (yes)	5309 (15.2)	2839 (15.7)	1372 (13.7)	421 (16.1)	677 (15.4)	< 0.001
History of dyslipidemia (yes)	6475 (18.5)	3292 (18.3)	1743 (17.5)	526 (20.1)	914 (20.8)	< 0.001
Family history of T2D	6781 (19.4)	3299 (18.3)	1953 (19.6)	571 (21.8)	958 (21.8)	< 0.001
Personal history of gestational diabetes	55 (0.2)	27 (0.1)	19 (0.2)	2 (0.1)	7 (0.2)	0.6

*BMI* body mass index, *STAI* state-trait anxiety inventory, *T2D* type 2 diabetes.

^a^Values represent n (%) unless noted otherwise.

^b^High trait anxiety was defined as STAI-T ≥ 40. STAI-T, form Y; score range: 20–80 points; higher scores reflect higher proneness to anxiety.

^c^Obtained by ANOVA or Chi-Squared test, as appropriate.

^d^ “Educational level” contained 1389 missing values that were imputed prior to the main analysis.

^e^"Without professional activity" includes "homemaker, disabled, unemployed or student; “Employment category” contained 1581 missing values that were imputed prior to the main analysis.

^f^Assessed with the International Physical Activity Questionnaire-Short Form according to established scoring criteria; “Physical activity level” contained 1592 missing values that were imputed prior to the main analysis; “Sedentariness” contained 1626 missing values that were imputed prior to the main analysis.

^g^ “Alcohol use” contained 1115 missing values that were imputed prior to the main analysis.

^h^”Smoking status” contained 1126 missing values that were imputed prior to the main analysis.

^i^Depressive symptomatology was defined as Center for Epidemiologic Studies-Depression Scale, CES-D ≥ 17 in men and CES-D ≥ 23 in women; the variable contained 1059 missing values that were imputed prior to the main analysis.

We tested the interaction between anxiety and insomnia as regards T2D risk. Next, using the method applied by Deschênes et al.^28^, the risk estimates of T2D in the different groups were compared to the “no anxiety or insomnia” group in the main model. In a secondary model, the risk estimates were compared to the “anxiety only” group, as anxiety is a more prevalent disorder than chronic insomnia in the general population^6,7^. These associations were investigated using Cox proportional hazards models, providing hazard ratios (HR) and 95% confidence intervals (CI). Given that the STAI-T completion date (baseline) varied across participants, age was used as a time scale. Participants contributed person-time from their age at baseline (i.e. age at which they completed STAI-T) to their age at T2D onset, age at the time of death (obtained from the CépiDC registry), age at the time of the last follow-up, or age as of April 29, 2022, whichever occurred first. The proportional hazards assumption of the Cox model was tested and confirmed with the rescaled Schoenfeld-type residuals method. The partially-adjusted models (Model 1) were controlled for age (time scale), sex, and obesity (BMI ≥ 30 kg/m^2^). The fully-adjusted models (Model 2) were adjusted for age (time scale), sex, education, employment, children aged < 18 years in the household, obesity (BMI ≥ 30 kg/m^2^), physical activity, sedentariness, alcohol consumption, smoking status, hypertension, dyslipidemia, family history of T2D, and depressive symptoms.

In a sensitivity analysis, we added total energy intake and proportion of the total energy from carbohydrates to model 2. In a second sensitivity analysis, we fit our main model restricting the sample to participants with ≥ 2 years of follow-up.

As differences in anxiety and insomnia prevalence according to sex have been highlighted in the literature^29,30^, we performed tests for interaction (significance level *p* < 0.10) by sex. The main tests were two-sided and *p* < 0.05 was considered as evidence for statistical significance. R version 4.1.2 (R Foundation, Vienna, Austria) was used for the analyses.

### Ethical standards

The NutriNet-Santé cohort study is conducted according to the Declaration of Helsinki guidelines. It was approved by the Institutional Review Board of the French Institute for Health and Medical Research (INSERM # 00000388FWA00005831) and by the National Commission on Informatics and Liberty (CNIL # 908450 and # 909216). NutriNet-Santé is registered at: https://clinicaltrials.gov/ct2/show/NCT03335644. Electronic informed consent was obtained from all volunteers prior to inclusion in the cohort.

## Results

**Figure 1:**
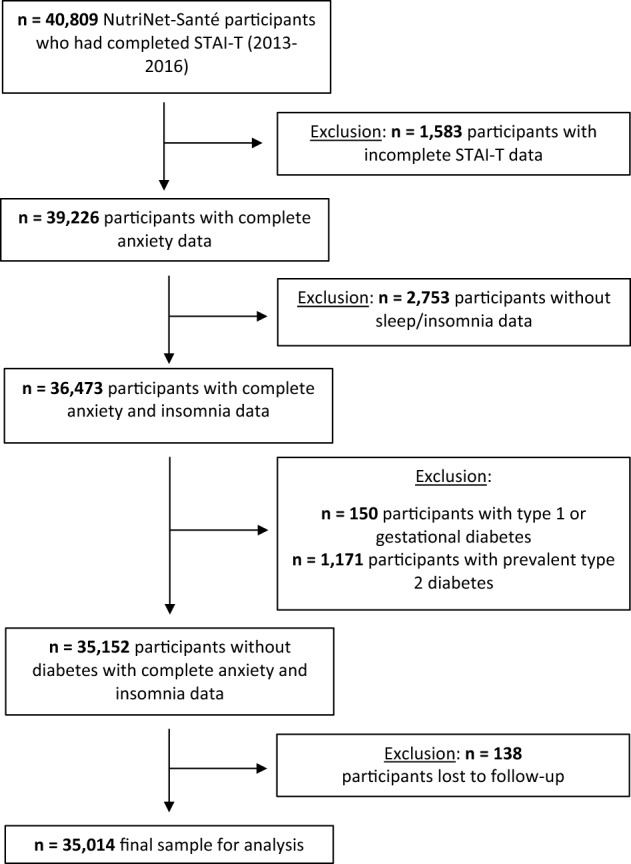
Participant selection flowchart.

A total of 40,809 participants in NutriNet-Santé completed STAI-T and were thus eligible for this analysis. Of these, 1583 had incomplete anxiety data and were excluded from the sample. Next, those who did not have sleep/insomnia data were ineligible for the study (n = 2753). Participants with type 1 diabetes (incident or prevalent), gestational diabetes or prevalent T2D at the time of completion of STAI-T were also excluded (n = 1321), as were those lost to follow-up (n = 138). Thus, the final sample was composed of 35,014 participants (76.2% women, mean age = 52.4 ± 14.0 years) (Fig. 1).

The mean follow-up time in this study was 5.9 ± 2.1 years. By the end of the follow-up, there were 378 (1.1%) incident T2D cases. Interaction tests by sex were not statistically significant (*p* > 0.2) thus baseline descriptive characteristics are presented in the full sample, according to morbidity group (Table 1).

The final sample was predominantly female, relatively well educated, non-smoking and with a normal-range BMI. The anxiety-insomnia group (n = 4386; 12.5% of the full sample) had the highest percentage of women, participants with depressive symptoms, history of hypertension, low physical activity and obesity. In that group, 1.5% developed T2D, which represented a greater proportion than in the other study groups (Table 1). The interaction tests between insomnia and anxiety regarding risk of T2D were not statistically significant (*p* > 0.3).

Table 2 presents Cox model results of the associations between the 4 morbidity groups and incident T2D, with the “no anxiety or insomnia” group as the reference. In Model 1 (adjusted for age, sex, and obesity), anxiety, unlike insomnia, emerged as a significant predictor of T2D (HR: 1.37; 1.07–1.76). In the fully-adjusted model (Model 2), high trait anxiety alone and chronic insomnia alone were not associated with incident T2D. However, participants with both high trait anxiety and chronic insomnia had an increased risk of incident T2D (HR: 1.40; 95% CI 1.01, 1.95) compared with participants without insomnia or anxiety.

**Table 2:** Prospective association between high trait anxiety, chronic insomnia, their comorbidity and incident type 2 diabetes mellitus (NutriNet-Santé cohort, France).

	Model 1		Model 2	
	HR [95% CI]	*p*-value	HR [95% CI]	*p*-value
No anxiety or insomnia	1.00	Ref	1.00	Ref
High trait anxiety^a^ alone	**1.37 [1.07–1.76]**	**0.015**	1.21 [0.93–1.57]	0.161
Chronic insomnia alone	1.07 [0.71–1.62]	0.731	0.96 [0.64–1.45]	0.855
Anxiety-insomnia comorbidity	**1.70 [1.27–2.28]**	** < 0.001**	**1.40 [1.01–1.95]**	**0.045**

Model 1: Cox model adjusted for age (time-scale), sex and obesity status (BMI > 30 kg/m^2^) (N = 35,014, n = 378 incident type 2 diabetes cases).

Model 2: Cox model adjusted for age (time-scale), sex, obesity status (BMI > 30 kg/m^2^), education, employment, children aged < 18 y in the household, physical activity, sedentariness, alcohol consumption, smoking status, hypertension, dyslipidemia, family history of type 2 diabetes mellitus, and depressive symptoms (N = 35,014, n = 378 incident type 2 diabetes cases).

*STAI* state-trait anxiety inventory.

Significant values are given in bold.

^a^High trait anxiety is defined as STAI-T ≥ 40.

To better understand the relationship between the anxiety-insomnia comorbidity and T2D and to test our secondary hypothesis, we compared participants having both disorders to those having only high trait anxiety. Table 3 presents these Cox model results. In Model 1, controlled for age, sex and obesity, participants with no anxiety or insomnia were significantly less likely to develop T2D during follow-up compared to participants with only high trait anxiety (HR = 0.73; 0.57–0.93). In Model 2, that associated was attenuated. Likewise, Model 2 revealed that participants with anxiety-insomnia comorbidity were not significantly more likely to develop T2D compared to participants with high trait anxiety alone.

**Table 3:** Prospective association between chronic insomnia, anxiety-insomnia comorbidity and incident type 2 diabetes compared with participants with high trait anxiety alone (NutriNet-Santé cohort, France).

	Model 1		Model 2	
	HR [95% CI]	*p*-value	HR [95% CI]	*p*-value
High trait anxiety^a^ alone	1.00	Ref	1.00	Ref
No anxiety or insomnia	**0.73 [0.57–0.93]**	**0.012**	0.83 [0.64–1.08]	0.160
Chronic insomnia alone	0.78 [0.51–1.20]	0.256	0.80 [0.52–1.23]	0.306
Anxiety-insomnia comorbidity	1.24 [0.91–1.69]	0.173	1.16 [0.85–1.59]	0.355

Model 1: Cox model adjusted for age (time-scale), sex and obesity status (BMI > 30 kg/m^2^) (N = 35,014, n = 378 incident type 2 diabetes cases).

Model 2: Cox model adjusted for age (time-scale), sex, obesity status (BMI > 30 kg/m^2^), educational, employment, children aged < 18 y in the household, alcohol consumption, smoking status, hypertension, dyslipidemia, family history of type 2 diabetes mellitus, and depressive symptoms (N = 35,014, n = 378 incident type 2 diabetes cases).

*STAI* state-trait anxiety inventory.

Significant values are given in bold.

^a^High trait anxiety is defined as STAI-T ≥ 40.

Supplementary Table S2 presents the results of the two sets of sensitivity analysis. In the first set (Model 3), we added to Model 2 covariates regarding total mean energy intake without alcohol (kcal/day) and proportion (%) of the total energy from carbohydrates. The addition of these data resulted in the reduction of the sample to 22,819 participants of whom n = 231 were incident T2D cases. The results were consistent with those obtained in the main analysis (HR = 1.61; 1.06–2.45).

In the second set of sensitivity analyses (Model 4), we fit the same models as those in the main analysis, while restricting our sample to participants with ≥ 2 years of follow-up (N = 32,376; n = 226 incident T2D cases). The results (Model 4) likewise remained consistent with those obtained in the main analysis.

## Discussion

As reduction of T2D rates is a public health priority, it is important to advance knowledge about modifiable risk factors for the disease in order to inform prevention efforts. We focused on mental health-related determinants and more specifically on the impact of the comorbidity between two prevalent mental disorders. To our knowledge, this prospective study is the first to provide estimates of the association between anxiety-insomnia comorbidity and incident T2D. It included 35,014 men and women, of whom 378 developed T2D during a mean follow-up of nearly 6 years. We employed the validated STAI-T tool for the assessment of trait anxiety and based our insomnia definition on DSM-5 and ICSD-3 criteria. We compared T2D risk across 4 morbidity groups: no anxiety or insomnia, anxiety alone, chronic insomnia alone, and anxiety-insomnia comorbidity. In the main analysis, high trait anxiety alone and chronic insomnia alone were not significantly associated with T2D. However, participants with anxiety-insomnia comorbidity were significantly more likely to develop T2D compared to those without anxiety or insomnia. Our hypothesis was therefore partially supported.

Our main findings regarding anxiety alone are in contrast with a recent meta-analysis which concluded that anxiety is a significant risk factor for T2D^9^. It should be noted that the 14 observational studies included in that meta-analysis addressed different types of anxiety (e.g., trait anxiety, generalized anxiety disorder, post-traumatic stress disorder, social phobia) rendering the methodology heterogeneous. However, null findings regarding anxiety as a predictor of T2D (measured with STAI-T on a continuous scale) were reported by Abraham et al. in the prospective Multi-Ethnic Study of Atherosclerosis^31^ included in the meta-analysis. In that meta-analysis, adjustment for depression did not have an impact on the estimated association between anxiety and T2D^9^. Likewise, our findings were independent of depressive symptoms. Depression has in fact been the most studied mental health determinant of T2D^32^, alone or together with other mental health conditions. For example, symptoms of depression, anxiety, and insomnia, were found to increase the risk of pre-diabetes and T2D in Swedish middle-aged men but not women^33^. Next, one large prospective study reported that the anxiety-depression comorbidity was a risk factor for incident T2D^28^. These authors revealed that participants with an anxiety-depression comorbidity had a higher risk of T2D than did participants with anxiety alone or depression alone^28^. Whereas anxiety and depression are related disorders, their distinctive features are numerous and important^5^.

Next, no association between chronic insomnia and incident T2D was found in our study. An umbrella review of two meta-analyses^10,11^ concluded that a positive association exists between baseline sleep disorders (i.e., difficulty maintaining sleep and difficulty initiating sleep) and T2D onset^12^. None of the studies included in the meta-analyses adjusted for anxiety^10,11^. Regarding insomnia itself, prospective studies that have investigated its association with incident T2D concluded to a positive association^14–16^. However, prior research has focused on populations with prediabetes, or has investigated insomnia without considering its impact on daily life^14,15^. Moreover, Green et al. observed that the association between insomnia and T2D was attenuated when psychiatric distress was viewed as a cofounding variable^14^.

As hypothesized, NutriNet-Santé participants with anxiety-insomnia comorbidity were more likely to develop T2D compared to their counterparts without anxiety or insomnia. This finding was reinforced by the sensitivity analyses. Given the frequent comorbidity of these two disorders^4^, some authors suggest that they might in fact represent different symptoms of the same pathology^34^. However, in our study, there was a significantly increased T2D risk when the reference group was ‘no anxiety or insomnia’ and a non-significant risk when the reference group was ‘anxiety alone’. When comparing the ‘anxiety-insomnia comorbidity’ group with the ‘no anxiety or insomnia’ group, there is a clearer difference in health status than when the comparison is made with the ‘anxiety alone’ group. In addition, our study is not based on clinical diagnoses of anxiety; it is also likely that individuals with the most severe symptoms were not included in the analysis^35^.

It has been estimated that anywhere from a quarter to about half of individuals with anxiety also have insomnia symptoms^36^. In addition, 36% of those with insomnia symptoms also have anxiety symptoms^37^. Uhde and Cortese suggest 3 theoretical models to explain this comorbidity^34^. First, anxiety and insomnia could represent a single mental health disorder; second, they could be separate conditions, one of which precedes and influences the development of the other; finally, they could be separate conditions caused by an independent factor^34^. Ohayon and Roth described this comorbidity according to the onset of the two disorders. Insomnia appeared before anxiety in 18% of cases, at the same time in 39%, and after anxiety in 44% of cases^38^, arguing for a complex, possibly bidirectional relationship between the two disorders^17,34^. At present, each disorder is regarded as having sufficiently distinct characteristics and is described individually in the DSM-5^5^.

Anxiety and insomnia have several common pathways impacting the central nervous system and metabolism and may represent mechanisms in the pathogenesis of T2D^17^. There is strong evidence for the role of the hypothalamus–pituitary–adrenal (HPA) axis in sleep regulation^39^. Also, stress activates the sympathetic nervous system and the HPA axis, resulting in the release of glucocorticoids, such as cortisol, leading to an increase in blood glucose concentrations^40^. In addition, excessive activation of the HPA axis induces sleep fragmentation, a stress-prone state, further increasing cortisol levels^17^. On the other hand, insomnia symptoms and anxiety disorders are also associated with markers of systemic inflammation^41^, such as increased C-reactive protein or interleukin-6, which are themselves associated with T2D^42^. In addition, anxiety and insomnia are associated with behavioral and lifestyle risk factors that have themselves been linked to T2D. For example, both disorders are strongly associated with weight gain and obesity^43,44^, and with physical inactivity and sedentariness^45^. Finally, cognitive mechanisms have also been explored, as anxiety is associated with decreased concentration, lack of motivation and impaired decision making, all of which favor certain unfavorable behaviors, such as reduced adherence to medical regimens^12^.

This study has some limitations. As NutriNet-Santé is focused on nutrition and health, it is possible that it attracts individuals interested in these topics. This may lead to an underestimation of studied exposure-outcome associations. Our sample included a relatively small number and percentage of participants with chronic insomnia alone, reducing the available statistical power. NutriNet-Santé participants are predominantly women (> 70%) of high socioeconomic status^23^, limiting generalizability of the findings. Next, as elsewhere around the world, T2D is generally underdiagnosed in France: it has been estimated that about 20% of cases remain undetected^46^. In our study, incident T2D cases were assessed and validated through multiple sources. Regarding our exposure variables, one-time subjective measures of anxiety and insomnia were used, although for the former we used a validated tool (STAI) and for the latter we followed the ICSD-3 and DSM-5 criteria. Also, even though the STAI-T is one of the most widely used tools in epidemiological research concerning anxiety, it cannot replace a complete clinical diagnosis. Finally, even though the analyses were adjusted for a number of potential cofounders, omitted variables such as other sleep variables (e.g., total sleep time, obstructive sleep apnea, etc.), other mental health conditions, family history of anxiety or insomnia disorders or ethno-racial status might have impacted the observed associations. Future studies could address questions of interaction, confounding, and/or mediation by such variables.

Nonetheless, this study presents several strengths. To our knowledge, it is the first study investigating the association between anxiety-insomnia comorbidity and incident T2D on a large scale. It uses a very large and diverse sample of French adults recruited from the general population. As our exposure variable was composed of 4 morbidity groups and the models allowed us to calculate separate T2D risk estimates. Next, the analysis was adjusted for well-established T2D risk factors, such as smoking, alcohol use, sedentariness and obesity^1^, among other covariates. Strengths also include the longitudinal design, with almost 6 years of follow-up, thus reverse causality is an unlikely explanation for the obtained results.

## Conclusion

In this large population-based sample, participants with anxiety-insomnia comorbidity were more likely to develop T2D than were participants with no anxiety or insomnia. Future prospective population-based studies are needed to confirm the findings. Given the high prevalence of anxiety and insomnia in the general population, the results highlight the need for prevention programs to address these mental health conditions before they lead to metabolic complications. Moreover, future analyses in specific subgroups (e.g., individuals with poor dietary quality, individuals with concurrent chronic physical and/or mental health conditions) could advance knowledge and help inform targeted T2D prevention efforts.

### Supplementary Information

Supplementary Tables.

## Data Availability

Data described in the manuscript can be made available upon request pending application and approval. Researchers from public institutions can submit a collaboration request including information on the institution and a brief description of the project to collaboration@etude-nutrinet-sante.fr. All requests are reviewed by the steering committee of the NutriNet-Santé study. A financial contribution may be requested. If the collaboration is accepted, a data access agreement will be necessary and appropriate authorizations from the competent administrative authorities may be needed. In accordance with existing regulations, no personal data will be accessible.

## Abbreviations

BMI: Body mass index
CES-D: Center for epidemiologic studies-depression scale
CI: Confidence interval
DSM-5: Diagnostic and statistical manual of mental disorders—fifth edition
HPA: Hypothalamus–pituitary–adrenal (axis)
HR: Hazard ratio
ICSD-3: International classification of sleep disorders—third edition
STAI: State-trait anxiety inventory
STAI-T: Trait anxiety subscale of the state-trait anxiety inventory
T2D: Type 2 diabetes mellitus
