# Supplementary material for: Chronic insomnia, high trait anxiety and their comorbidity as risk factors for incident type 2 diabetes mellitus
Source: Sci Rep. 2024 May 24;14:11927. doi: 10.1038/s41598-024-62675-y (PMC11126668; doi:10.1038/s41598-024-62675-y)
Supplement: Supplementary file 1 — Supplementary Tables. [file 41598_2024_62675_MOESM1_ESM.docx]

Ref: Submission ID 4de4dc00-6141-4243-b5bc-fb13f0a3eefe

Manuscript: Chronic insomnia, high trait anxiety and their comorbidity as risk factors for incident type 2 diabetes

Authors: Duquenne et al.

**Supplementary Table S1: Type 2 diabetes (T2D) case ascertainment in NutriNet-Santé**

| NutriNet-Santé study | Participants report major health events and all current medication and treatment use (a search engine of the *Vidal* drug database is used to facilitate medication data entry):   - On the yearly health questionnaire - On a specific health status questionnaire every 6 months - At any time using a dedicated platform on the study website   Questions asked:   - “Have you been diagnosed with type 2 diabetes (if yes, please indicate the date of diagnosis)” - “Are you treated for type 2 diabetes?” |
| --- | --- |
| French National Health Insurance Database (SNIIRAM) | Provides detailed information about reimbursement of prescription medication, medical consultations and hospitalizations.  Cross-referencing with the SNIIRAM database confirmed > 80% of the cases (ICD-10 code E11). |
| ATC codes | ATC codes considered for type 2 diabetes medication use were as follows:  A10AB01, A10AB03, A10AB04, A10AB05, A10AB06, A10AC01, A10AC03, A10AC04, A10AD01, A10AD03, A10AD04, A10AD05, A10AE01, A10AE02, A10AE03, A10AE04, A10AE05, A10AE30, A10BA02, A10BB01, A10BB03, A10BB04, A10BB06, A10BB07, A10BB09, A10BB12, A10BD02, A10BD03, A10BD05, A10BD07, A10BD08, A10BD10, A10BD15, A10BD16, A10BF01, A10BF02, A10BG02, A10BG03, A10BH01, A10BH02, A10BH03, A10BX02, A10BX04, A10BX07, A10BX09, A10BX10, A10BX11, and A10BX12. |
| Clinical examination | Among participants who provided a blood sample at the clinical examination (2011-2014), 85.3% of those with elevated fasting blood glucose (i.e., >1.26 g/L) had consistently reported a diagnosis of type 2 diabetes and/or type 2 diabetes medication use. Elevated blood glucose without any concurrent self-report of type 2 diabetes diagnosis or treatment was not considered sufficiently specific to classify the participant as a type 2 diabetes case. |

**Supplementary table S2**: Sensitivity analysis of the prospective association between high trait anxiety, chronic insomnia, their comorbidity and incident type 2 diabetes, with the addition of diet-related covariates (Model 3) and following exclusion of participants with <2 y of follow-up (Model 4) (NutriNet-Santé cohort, France)

|  | **Model 3** |  | **Model 4** | |  |
| --- | --- | --- | --- | --- | --- |
|  | **HR [95% CI]** | **p** | **HR [95% CI]** | **p** | |
| No anxiety or insomnia | *1.00* | *ref* | *1.00* | *ref* | |
| High trait anxiety alone^1^ | 1.28 [0.91-1.78] | 0.154 | 1.38 [0.98-1.94] | 0.063 | |
| Chronic insomnia alone | 1.11 [0.66-1.87] | 0.690 | 0.97 [0.56-1.67] | 0.900 | |
| Anxiety-insomnia comorbidity | **1.61 [1.06-2.45]** | **0.027** | **1.74 [1.15-2.62]** | **0.009** | |

STAI: State-Trait Anxiety Inventory

^1^High trait anxiety is defined as STAI-T ≥ 40

Model 3: Cox model adjusted for age (time-scale), sex, obesity status (BMI > 30 kg/m^2^), educational level, employment category, children aged < 18 y in the household, alcohol consumption, smoking status, hypertension, dyslipidemia, family history of type 2 diabetes, depressive symptoms, total energy intake without alcohol (kcal), and proportion of energy from carbohydrates (N = 22,819; n=231 incident type 2 diabetes cases, mean follow-up: 6.2 ± 2.1 years)

Model 4: Cox model adjusted for age (time-scale), sex, obesity status (BMI > 30 kg/m^2^), educational level, employment category, children aged < 18 y in the household, alcohol consumption, smoking status, hypertension, dyslipidemia, family history of type 2, depressive symptoms (N = 32,376; n=226 incident type 2 diabetes cases, mean follow-up: 6.3 ± 1.7 years)
